# Cellular consequences of non-ablative radiotherapy: A novel approach to ventricular tachycardias

**DOI:** 10.1093/europace/euag282

**Published:** 2026-10-17

**Authors:** Claudia Maniezzi, Stefano Busti, Angela S Maione, Elena Sommariva, Chiara Florindi, Francesca Misitano, Ilenia Anzaldi, Francesco P Cammarata, Giusi I Forte, Roberto Catalano, Gaetano M De Ferrari, Francesco Lodola, Antonio Zaza

**Affiliations:** Department of Biotechnology and Bioscience, University of Milano-Bicocca, p.zza della Scienza 2, Milan 20126, Italy; Department of Biotechnology and Bioscience, University of Milano-Bicocca, p.zza della Scienza 2, Milan 20126, Italy; iPSC Facility for Cardiac Modelling and Function, Centro Cardiologico Monzino IRCCS, Milan, Italy; Unit of Inherited Cardiomyopathies, Centro Cardiologico Monzino IRCCS, Milan, Italy; Department of Biotechnology and Bioscience, University of Milano-Bicocca, p.zza della Scienza 2, Milan 20126, Italy; Department of Biotechnology and Bioscience, University of Milano-Bicocca, p.zza della Scienza 2, Milan 20126, Italy; Department of Biotechnology and Bioscience, University of Milano-Bicocca, p.zza della Scienza 2, Milan 20126, Italy; Institute of Bioimaging and Complex Biological Systems, National Research Council, Cefalù, Italy; National Institute for Nuclear Physics, Laboratori Nazionali del Sud, Catania, Italy; Institute of Bioimaging and Complex Biological Systems, National Research Council, Cefalù, Italy; National Institute for Nuclear Physics, Laboratori Nazionali del Sud, Catania, Italy; National Institute for Nuclear Physics, Laboratori Nazionali del Sud, Catania, Italy; Unit of Cardiology, Department of Medical Sciences, University of Turin, Turin, Italy; Division of Cardiology, Molinette Hospital, Città della Salute e della Scienza, Turin, Italy; Department of Biotechnology and Bioscience, University of Milano-Bicocca, p.zza della Scienza 2, Milan 20126, Italy; Department of Biotechnology and Bioscience, University of Milano-Bicocca, p.zza della Scienza 2, Milan 20126, Italy

**Keywords:** Non-ablative radiotherapy, Na^+^ current, ROS, CaMKII

## Abstract

**Aims:**

Radiotherapy (RT) with a single application of ionizing radiation [Stereotactic Arrhythmia Radioablation (STAR)] has been suggested as a non-invasive alternative to radiofrequency in ablating ventricular tachycardia (VT). Emerging data reveal that STAR may suppress VT without substrate destruction by enhancing impulse conduction instead, through increased expression of NaV1.5 channels and connexin 43. The study aims to investigate electrophysiology and intracellular Ca^2+^ dynamics in cardiomyocytes from mice subjected to *in vivo* RT. The emerging data led us to evaluate biochemical changes potentially linking electrophysiological response to ionizing irradiation.

**Methods and results:**

Cardiomyocytes isolated 2 weeks after RT with low-dose (15 Gy) or high-dose (25 Gy) were compared to those of sham-treated mice (CTRL). We evaluated: (i) the properties of the transient (*I*_NaT_) and sustained (*I*_Nasus_) components of *I*_Na_; (ii) action potential (AP) parameters, including the prevalence of early afterdepolarizations (EADs); (iii) intracellular Ca^2+^ dynamics; (iv) CaMKII phosphorylation, and (v) reactive oxygen species (ROS) content. 25 Gy RT (i) increased *I*_NaT_ and, to a larger extent, *I*_Nasus_ (increased *I*_Nasus_/*I*_NaT_ ratio); (ii) increased AP amplitude, +*dV*/*dt*_MAX_ and duration (APD) and facilitated EADs; (iii) depressed intracellular Ca^2+^ dynamics. 15 Gy RT had similar but smaller effects (dose-dependency). 25 Gy RT reduced CaMKII phosphorylation but increased cell ROS content, thus providing a mechanism for the late component of *I*_Na_ (*I*_NaL_) enhancement.

**Conclusion:**

The results support the view that STAR may suppress VT by increasing conduction velocity, with APD prolongation providing an additional mechanism. On the other hand, *I*_Nasus_ enhancement and Ca^2+^ handling depression may impair electrical stability and contractility in the irradiated region.

## Graphical Abstract

**Graphical Abstract:**
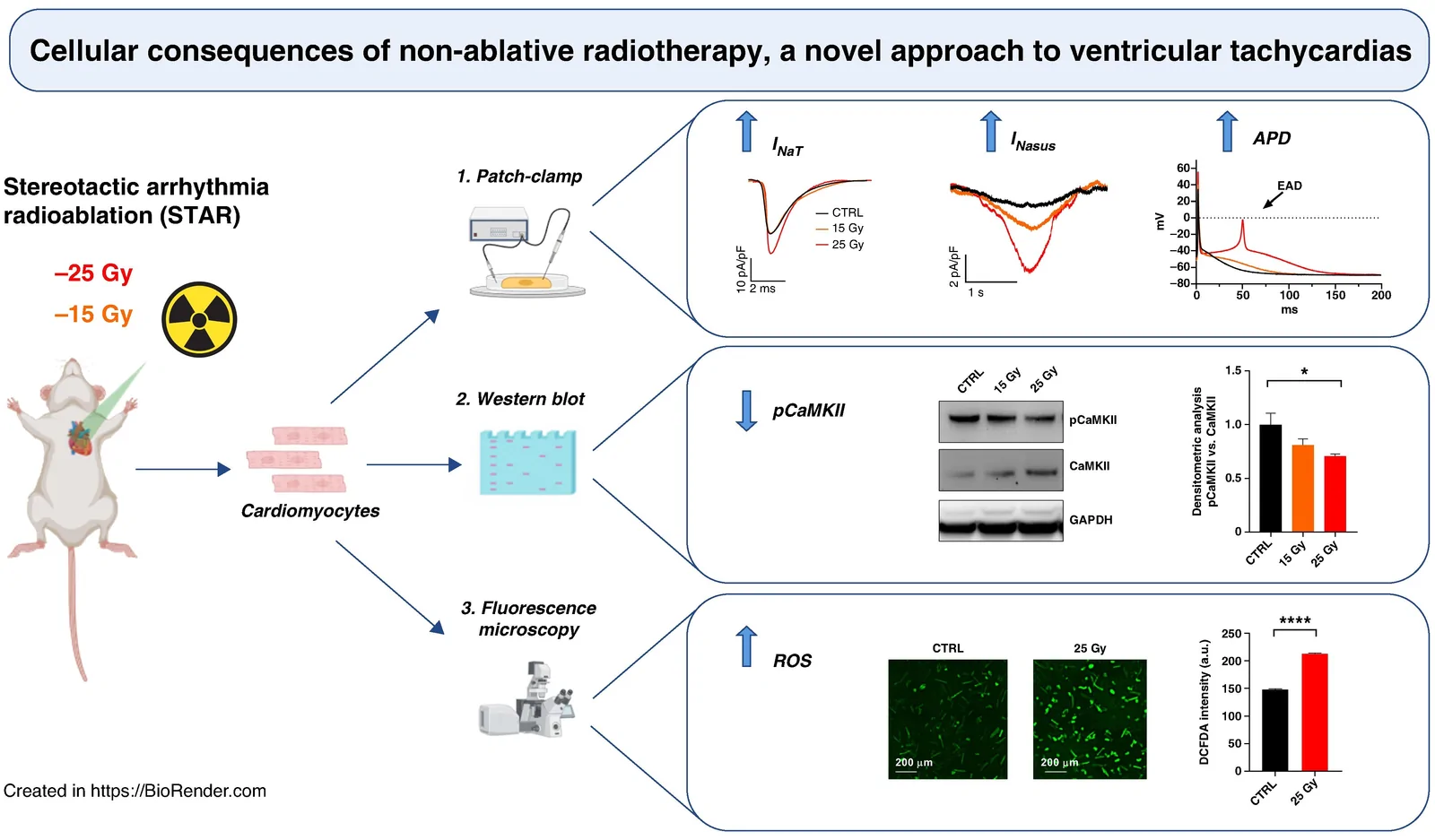

## Introduction

Ventricular tachycardias (VTs) are a frequent complication of advanced structural heart disease because of a complex combination of anatomical and functional predisposing factors. Besides being a life-threatening condition, VTs may be symptomatic and, due to the risk of inducing syncope, a strong limitation to patients’ lifestyle.

Ventricular tachycardias treatment often requires anatomical ablation of the arrhythmogenic substrate, mostly a re-entry circuit, performed by local application of thermal energy (radiofrequency) or, since recently, of non-thermal one (pulsed-field).^1^ Albeit useful, the procedure is invasive, particularly in the case of epicardial ablation^2^ major complications occur in up to 5–6% of procedures^3^ and VT recurrence rate is between 27% and 55% depending on the substrate.^4^ Furthermore, the scar generated by the procedure might provide a new arrhythmogenic substrate.

External application of ionizing radiation (radiotherapy, RT) to ablate the VT substrate has been proposed in 2015 as a non-invasive alternative to catheter ablation^5^ and has been evaluated in small clinical studies with positive and enduring results.^6–9^ This approach, named Stereotactic Arrhythmia Radioablation (STAR), is based on a single delivery of high-dose (25 Gy) RT to a relatively small volume (mean 25 cc) of myocardium by a focused beam.

As conceived, STAR is still ‘ablative’, i.e. meant to replace the tissue supporting the arrhythmia with an unexcitable fibrotic scar. Nonetheless, VT suppression was observed at times after RT delivery hardly compatible with scar formation,^6^ thus suggesting a functional mechanism instead. A recent article by Zhang et al.^10^ indeed reported the absence of fibrosis in the irradiated tissue from human hearts in which VT was successfully treated by 25 Gy RT. When applied to mice, the irradiation increased myocardial conduction velocity enhanced the expression of Na^+^ channel proteins (NaV1.5) and of the gap-junctional protein Cx43. This finding opens the theoretically plausible possibility to treat VT by boosting impulse propagation, as opposed to discontinuing it by tissue damage. Since this approach aims to preserve viability of irradiated myocytes, its effects on myocyte electrophysiology, ionic homeostasis and biology deserve investigation.

Enhanced expression of NaV1.5 channel protein expectedly increases the Na^+^ current (*I*_Na_); however, evidence on this aspect is missing. Moreover, whether the transient (*I*_NaT_) and sustained (*I*_Nasus_) components of *I*_Na_ are equally affected is of interest. Indeed, whereas *I*_NaT_ upregulation is crucial for conduction improvement, *I*_Nasus_ enhancement may affect both membrane potential and intracellular Ca^2+^ homeostasis, with undesirable effects on electrical stability, tissue remodelling, and energetic competence.^11^

In the present study we investigate the chronic effects (14 days from RT) of *in vivo* RT on murine cardiomyocytes (CMs) with the aims to (i) test whether *I*_Na_ was modulated, (ii) compare the response of *I*_Nasus_ vs. *I*_NaT_, and (iii) test whether *I*_Na_ changes affected action potential (AP) contour. After observing that RT markedly enhanced *I*_Nasus_, we proceeded to evaluate RT effect on intracellular Ca^2+^ dynamics, notoriously influenced by Na^+^ influx, along with reactive oxygen species (ROS) content and CaMKII activation, known factors in *I*_Nasus_ upregulation. Two RT doses were evaluated: 25 Gy, used in previous studies demonstrating STAR efficacy on VT; 15 Gy to establish whether the changes responsible for VT suppression could be achieved with a lower dose.

## Methods

### Data availability

All data that support the findings of this study are available from the corresponding author on reasonable request.

### Animal model

Six to eight–weeks-old male CD-1 mice (Charles River Laboratory), weighing 20 ± 3 g were enrolled and divided into three groups: (i) untreated, (ii)15 Gy, and (iii) 25 Gy-treated mice. Animals were housed in individual IVC cages with 3 subjects per cage, at constant temperature (23°C–25°C) under a 12/12 h light/dark cycle with *ad libitum* access to food and water. The animals were randomly divided into eight experimental sessions following a specific workflow. After 1 week of acclimatization, radiation treatment was administered by anesthetizing the animals with a mixture of isoflurane and oxygen. Then, mice were evaluated and monitored to prevent acute toxicity and distress. Four days after treatment, they were moved to the physiology laboratory, where they remained one week for acclimatation. A total of 14 days elapsed between RT and the start of post-treatment analyses. Efforts were made to replace, to reduce and to refine the use of laboratory animals. Except for one mouse, which died due to anaesthesia, all the enrolled animals were successfully irradiated, showing no acute toxicity. All procedures were performed in accordance with the European Communities Council directive and Italian regulations (EEC Council 2010/63/EU and Italian D.Lgs. 26/2014). The project was approved by the Italian Ministry of Health (authorization number n. 985/2024-PR).

### Irradiation set-up

The animal irradiations were performed using the X-ray irradiation facility installed at the National Laboratory of the South of the National Institute of Nuclear Physics (LNS-INFN). The system is based on a high-performance X-ray tube (Varex Imaging, model HPX-320-11) capable of generating bremsstrahlung photons with a maximum peak voltage of 320 kV and a maximum current of ∼13.5 mA, with focal spot sizes of 0.4 and 1.0 mm (EN 12543 standards) and a maximum continuous power of 1800 W. The irradiation set-up includes a custom mechanical positioning system providing sub-millimetre accuracy through three degrees of freedom, together with a dedicated collimation system and light field for accurate beam alignment. A removable brass flattening filter was used to homogenize the beam profile, producing radiation fields with a uniformity better than ±5%. The entire system is remotely controlled and integrated with the radiation protection interlock system of the LNS-INFN.

In the present study, irradiations were performed using square radiation fields with a side length of 15 mm at a focus-to-surface distance of 50 cm. The X-ray tube was operated at the maximum voltage of 320 kV with the inherent 4 mm beryllium filtration, corresponding to a half-value layer of ∼1.3 mm Cu and an effective photon energy of about 88 keV.

The dosimetric characterization of the beam was carried out following the recommendations of the AAPM TG-61 protocol for kilovoltage X-ray beams. Measurements were performed using EBT4 Gafchromic films and a PTW 60019 microDiamond detector, whose single-crystal intrinsic diamond sensitive layer (∼1 μm thick) and very small, sensitive volume (∼0.004 mm^3^, radius 1.1 mm) provide high spatial resolution and near-tissue equivalence, making it particularly suitable for small-field dosimetry. To reproduce the scattering conditions typical of small animal irradiations, measurements were conducted in a small (6 × 6 × 6 cm^3^) RW3 water-equivalent phantom. The use of this phantom, together with the high-resolution detectors adopted, allowed accurate determination of beam profiles, flatness, symmetry, and output factors despite the challenges associated with small-field dosimetry, such as the lack of charged particle equilibrium and potential partial-volume effects in conventional detectors.

Several irradiation configurations, including single-field and multiple-field arrangements, were evaluated through dosimetric measurements to optimize the dose delivered to the heart and surrounding organs at risk. Based on these analyses, a single antero-posterior field with a size of 15 × 15 mm^2^ was selected as the optimal configuration. The measured beam profiles showed optimal flatness and symmetry, with the 95% isodose widths fully covering the target dimensions considered in this study. The overall uncertainty in the absorbed dose determination at the reference depth (*D_w_*_,*z*_ = 0) was estimated to be within ∼3.5%, confirming the reliability of the adopted dosimetric methodology.

### Electrophysiology

Mouse myocytes (CMs) were studied 2 weeks after whole-heart irradiation (25 and 15 Gy) and compared to non-irradiated littermates (controls, CTRL). Ventricular myocytes were enzymatically isolated (see Supplementary material for details) and studied by patch-clamp (whole-cell configuration) after Ca^2+^ adaptation. Cells were superfused with Tyrode's solution (36.5°C) containing (mM): NaCl 154, KCl 4, CaCl_2_ 1.8, MgCl_2_ 1, HEPES 5, glucose 5.5, pH = 7.35 titrated with NaOH. The pipettes (tip resistance of 2–3 MΩ) were filled with a solution containing (mM): K-Aspartate 110, KCl 23, MgCl_2_ 3, CaCl_2_ 0.04, EGTA KOH 0.1, HEPES KOH 5, Na-CP 5, Na-ATP 5, Na-GTP 0.4, pH = 7.2 with KOH.

Current and voltage signals were sampled at 20 KHz and low-pass filtered at 2 or 10 KHz (for *I*_NaT_ and *I*_Nasus_ respectively).

Cell capacitance (*C*_m_) and series resistance were automatically estimated in each cell by the amplifier, based on minimization of the capacitive current transient under the whole-cell condition. *C*_m_ values are reported in Supplementary material online, *Table S2*.

#### Action potential recordings

Action potentials were recorded (I-clamp) during steady stimulation at 1 Hz (2 ms pulses at twice the threshold through the pipette). The following AP parameters were measured: diastolic potential (*E*_diast_), action potential amplitude (APA), maximal upstroke velocity (+*dV*/*dt*_MAX_), action potential duration at 90% repolarization (APD_90_). The prevalence of early afterdepolarizations (EADs), arrhythmogenic repolarization abnormalities typically associated with *I*_Nasus_ enhancement, was measured as the % of cells displaying them. Finally, to verify *I*_Nasus_ involvement in AP changes, AP response to selective *I*_Nasus_ blockade by TTX was assessed. To this end, 1 µM TTX (blocking *I*_Na_ by about 50%) was used to preserve excitability, which is a prerequisite in AP measurements.

#### Na^+^ current recording

*I*_Na_ transient (*I*_NaT_) and sustained (*I*_Nasus_) components were measured (V-clamp) within the same myocyte to obtain reliable *I*_Nasus_/*I*_NaT_ ratios. To this end, a single voltage step from −80 to 0 mV (*I*_NaT_ measurement) was followed by a voltage ramp of velocity mimicking that of repolarization (56 mV/s) (*I*_Nasus_ measurement). Such a step-ramp protocol was applied at physiological bath Na^+^ concentration (154 mM) and temperature (36.5°C), as required to resolve *I*_Nasus_ accurately; however, this resulted in large *I*_NaT_ amplitudes, potentially leading to underestimated *I*_NaT_ and its increment even after series (‘access’) resistance compensation (see Limitations). *I*_Na_ was measured, by subtraction, as the current sensitive to 30 µM TTX. *I*_NaT_ inactivation time constant (τ_h_) was estimated by exponential fitting of current decay during the depolarizing step. The equation of the mono-exponential function is *f*(*t*) = A*e^−^*^t^*^/τ^ + *C*.

The voltage ramp yielded quasi-steady-state *I*_Nasus_  *I*/*V* curves; the *I*_Nasus_ reversal potential (*E*_rev_) was obtained by linear fitting of the ascending portion of the *I*/*V* curve. *I*_Nasus_ was expressed as the ‘mean current’ recorded during the ramp, calculated as the *I*_Nasus_ integral divided by ramp duration. After checking that *I*_NaT_ inactivation course (τ_h_) was unchanged by RT (see Supplementary material online, *Table S2*), in the sake of uniformity we used the ‘mean’ value of the current in computing the *I*_Nasus_/*I*_NaT_ ratio. In the step-ramp protocol current traces were averaged over five repetitions applied at a 4 s interval.

In all protocols, *I*_Na_ was recorded under 80% compensation of access resistance (originally 5 ± 1 MΩ) thus resulting in an estimated bias of about 1 mV per each nA of *I*_Na_ difference between conditions. Liquid junction potential was uncompensated. Current density was obtained by normalization to *C*_m_, automatically estimated by the compensation circuit in each cell.

### Intracellular Ca^2+^ dynamics

Intracellular Ca^2+^ activity was measured by dynamic epifluorescence under field stimulation at constant rate (1 Hz) and physiological temperature. To this end, myocytes were loaded with the non-ratiometric Ca^2+^-sensitive (Fluo-4 AM, 10 μM) fluorophore. The following parameters of Ca^2+^ transients (CaT) were quantified: (i) CaT amplitude, (ii) CaT time to peak (*t*_peak_), (iii) maximal rate of CaT rise (*d*Ca/*dt*_MAX_), (iv) CaT decay kinetics (τ decay, estimated by exponential fitting of transient decay with the equation *f*(*t*) = A*e^−^*^t^*^/τ^ + *C*), and v) diastolic Ca^2+^ (CaD). Rate dependency of CaT properties was tested by incrementing the pacing rate from 1 to 5 Hz. Ca^2+^ fluorescence (*F*) at any timepoint was normalized to the value measured in the cell after prolonged quiescence (*F*0) and expressed in *F*/*F*0 units. Particularly for what concerns CaD comparison between different cells, this may provide only a qualitative estimate of the intervention effect. SR Ca^2^⁺ content (CaSR) was assessed in V-clamped CMs by integrating the caffeine (10 mM)-induced membrane current, which is mainly mediated by the Na⁺/Ca^2^⁺ exchanger (*I*_NCX_). Before caffeine, a loading train of V steps (−40 to 0 mV at 1 Hz) was applied.^12^

### Biochemical assays

1. CaMKII activation was assessed by western blot of homogenates of cell pellets from ventricles, evaluating the ratio between phosphorylated or oxidized CaMKII and the total CaMKII. Densitometric analysis was performed by ImageJ software (see Supplementary material for detail).
2. Intracellular ROS concentration was measured in single cells after incubation with the fluorescent probe 2′7′-dichclorofluorescin diacetate (DCFDA, 10 μM). Brightfield and fluorescence images were acquired with a Leica THUNDER LIVE imaging system (LAS X software) using a ×20 objective in a controlled environment (37°C, 5% CO_2_). Cell segmentation (identification of single cells) was performed on brightfield images by using a custom version of the Cellpose cyto3 model^13^ specifically trained to recognize CMs (efficiency >95%). Live/dead cells were distinguished (and excluded from ROS quantification) based on their different morphology by using the ilastik machine-learning classifier.^14^ Fluorescence intensities were extracted for each single cell by using Fiji/ImageJ.

### Experimental design

An ‘unpaired sample’ design was adopted for comparing the effects of irradiation vs. non-irradiation (control) and 15 vs. 25 Gy irradiation.

### Statistical analysis

Statistical analysis was carried out with GraphPad Prism 8 and R softwares.

Normality of distribution was assessed using D’Agostino–Pearson's normality test. One-way ANOVA (Dunnett's *post hoc* correction) or Kruskal–Wallis (Dunn's *post hoc* correction) was used for multiple comparisons. In the case of repeated measurements (e.g. rate dependency). Two-way ANOVA (Dunnett's *post hoc* correction) containing ‘Treatment’ (CTRL, 15 Gy, 25 Gy) and ‘Variable’ (pacing rate) factors was used. Significance of ‘Treatment × Variable interaction’ (i.e. difference between Treatments in their response to the Variable) was first tested; in its absence, significance of difference between Treatments at all Variable values was tested. Fisher’s exact test was applied to contingency tables for multiple comparison of categorical variables (EADs prevalence); the Benjamini–Hochberg correction was applied for *post hoc* comparisons between individual frequencies.

In figures, data are presented as mean ± SEM. For each experiment, the number of preparations or cells (*n*) and the number of animals from which they were obtained (*N*) are indicated in the respective figure legend.

## Results

### Effect of RT on *I*_Na_ components

The effect of 25 Gy RT on *I*_Na_ (*I*_NaT_ + *I*_Nasus_) was measured by the step-ramp protocol illustrated in *Figure 1A*. 25 Gy RT increased both peak *I*_NaT_ (+65%, *P* < 0.05, *Figure* *1B* and *C*) and *I*_Nasus_ (+242%, *P* < 0.0001, *Figure 1D–F*) densities. RT did not affect *I*_NaT_ inactivation course (see Supplementary material online, *Table S2*). Besides increasing maximal *I*_Nasus_ density, irradiation (at both 15 and 25 Gy) shifted by about +10 mV the potential at which maximal current was achieved, thus increasing current amplitude preferentially at potentials positive to −30 mV (*Figure 1E*). The *I*_Na_ ‘window’ encompasses potentials negative to −30 mV^15,16^; therefore, the finding suggests that the increase in *I*_Nasus_ was contributed by its late component (*I*_NaL_). The % increments in *I*_NaT_ and *I*_Nasus_ differed, thus resulting in a significant increase in the *I*_Nasus_/*I*_NaT_ ratio (+232%, *P* < 0.0001 *Figure 1G*). *I*_Nasus_  *E*_rev_ was +45.6 ± 1.8 mV in control and was unchanged by irradiation (*Figure 1H*), thus confirming that the current was carried by Na^+^ in all the experimental conditions.

**Figure 1:**
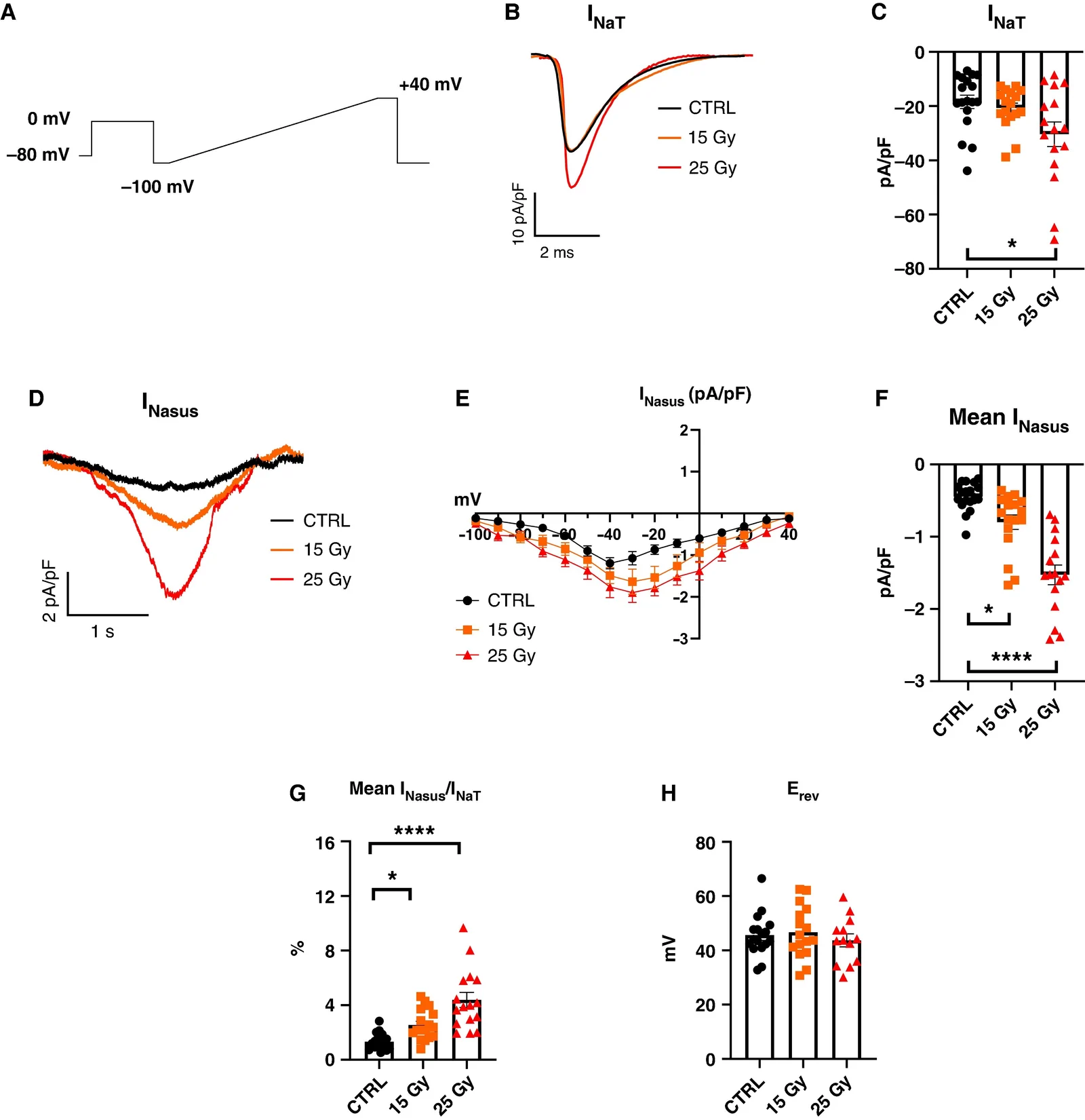
Effect of high dose (25 Gy) and low dose (15 Gy) RT on transient (*I*_NaT_) and late (*I*_Nasus_) component of *I*_Na_. (*A*) Step-ramp protocol. (*B–C*) Representative traces of *I*_NaT_ and quantification. (*D–F*) Representative traces of *I*_Nasus_, *I–V* relationship and mean *I*_Nasus_ quantification. (*G*) Mean *I*_Nasus_/*I*_NaT_ ratio. (*H*) Reversal potential (*E*_rev_). CTRL: *n* = 18, *N* = 3; 15 Gy: *n* = 17, *N* = 5; 25 Gy: *n* = 16, *N* = 2. Data are expressed as mean ± SEM. One-way ANOVA followed by Dunnett's *post hoc* test (*G* and *H*) and Kruskal–Wallis followed by Dunn's *post hoc* test (*C* and *F*), *P < 0.05, **P < 0.01, ***P < 0.001, ****P < 0.0001.

With 15 Gy, the changes were smaller (dose-dependency) and achieved statistical significance for *I*_Nasus_ only (+79%, *P* < 0.05, *Figure 1F*), thus increasing the *I*_Nasus_/*I*_NaT_ ratio (*Figure 1G*).

Mean *C*_m_ did not significantly differ between the experimental groups (see Supplementary material online, *Table S2*). Albeit changes in current density are, by definition, independent of cell size (or *C*_m_), absolute *I*_NaT_ amplitude differently correlated with *C*_m_ under the various experimental conditions (see Supplementary material online, *Figure S1*). The robust correlation found in non-irradiated hearts (*R*^2^ = 0.82, *P* = 0.0001) was partially lost with irradiation (*R*^2^ = 0.2935, *P* = 0.025 at 15 Gy and *R*^2^ = 0.5226, *P* = 0.0016) (see Supplementary material online, *Figure S1*). On the other hand, *I*_Nasus_ amplitude failed to correlate significantly with *C*_m_ in all experimental groups (see Supplementary material online, *Figure S2*).

Overall, the results show an *I*_NaT_ increment compatible with the effect of irradiation on myocardial conduction velocity,^10^ but also a preferential increase in *I*_NaL_, of potentially pathogenetic relevance.

### Effect of RT on the action potentials

Action potential parameters were measured at steady state during 1 Hz stimulation (*Figure 2*). 25 Gy RT enhanced APA (+12%, *P* < 0.05, *Figure 2B*) and +*dV*/*dt*_MAX_, (+38% *P* < 0.05, *Figure 2C*), consistent with the increment in *I*_NaT._ It also significantly prolonged APD_90_ (+110%, *P* < 0.01, *Figure 2D*), as expected from the increment in *I*_Nasus_; *E*_diast_ remained unchanged (*Figure 2E*). 15 Gy RT failed to modulate APA (*Figure 2B*) and +*dV*/*dt*_MAX_ (*Figure 2C*) but significantly prolonged APD_90_ (+69%, *P* < 0.05, *Figure 2D*). EADs prevalence was 12% in control CMs, 29% in 15 Gy-irradiated CMs, and 37% in 25 Gy-treated CMs (*Figure 2F*). Despite its dose-dependency, the increment in EADs prevalence failed to achieve statistical significance (*P* > 0.05 at Fisher’s test). EADs occurred at a membrane potential around −40 mV (plateau phase, *Figure 2A*), where *I*_Nasus_ was maximal (see *Figure 1E*), thus suggesting its direct contribution to EADs.

**Figure 2:**
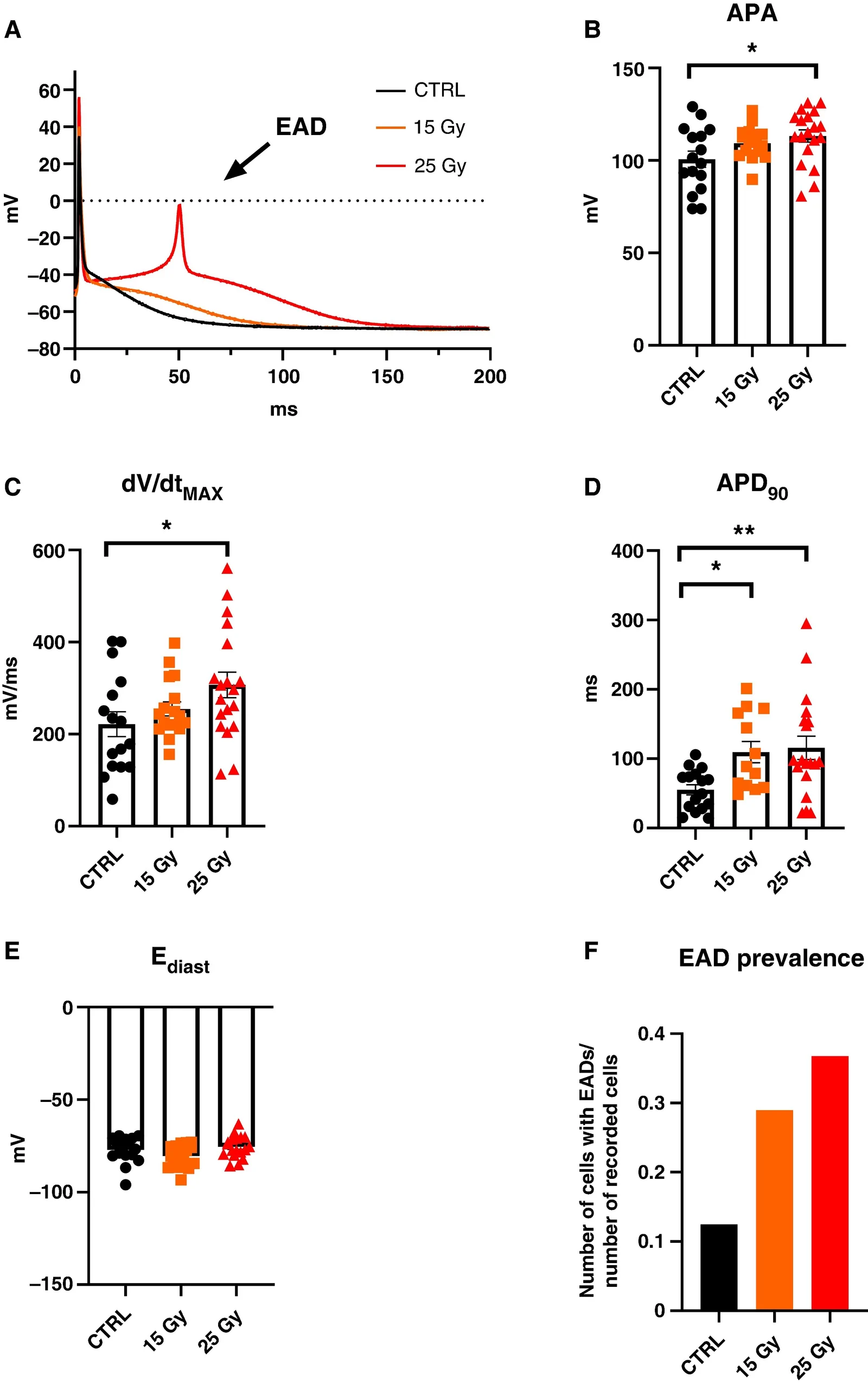
Effect of 15 and 25 Gy RT on APs. (*A*) Representative traces of AP recorded during stimulation (through the pipette) at 1 Hz. Early afterdepolarization (EAD) indicated by arrow. (*B*) AP amplitude (APA). (*C*) Maximal upstroke velocity (*dV*/*dt*_MAX_). (*D*) AP duration at 90% repolarization (APD_90_). (*E*) Diastolic potential (*E*_diast_). (*F*) EAD prevalence. CTRL: *n* = 16, *N* = 3; 15 Gy: *n* = 17, *N* = 3; 25 Gy: *n* = 19, *N* = 3. Data are expressed as mean ± SEM. One-way ANOVA followed by Dunnett's *post hoc* test (*B–E*) and Fisher’s followed by Benjamini–Hochberg *post hoc* test (*F*), *P < 0.05, **P < 0.01, ***P < 0.001, ****P < 0.0001.

To test whether *I*_Nasus_ enhancement contributed to repolarization slowing, we investigated the impact of selective *I*_Nasus_ blockade (by 1 µM TTX) on APD_90_ (*Figure 3*). TTX shortened APD_90_ more in RT CMs (−11% in 15 Gy and −25% in 25 Gy) than in CTRL ones (−6%), thus confirming the role of *I*_Nasus_ modulation on APD_90_ prolongation by RT. Notably, 1 µM TTX blocks *I*_Na_ by about 50%; stronger *I*_Na_ blockade might shorten APD further, but it would eventually prevent AP induction.

**Figure 3:**
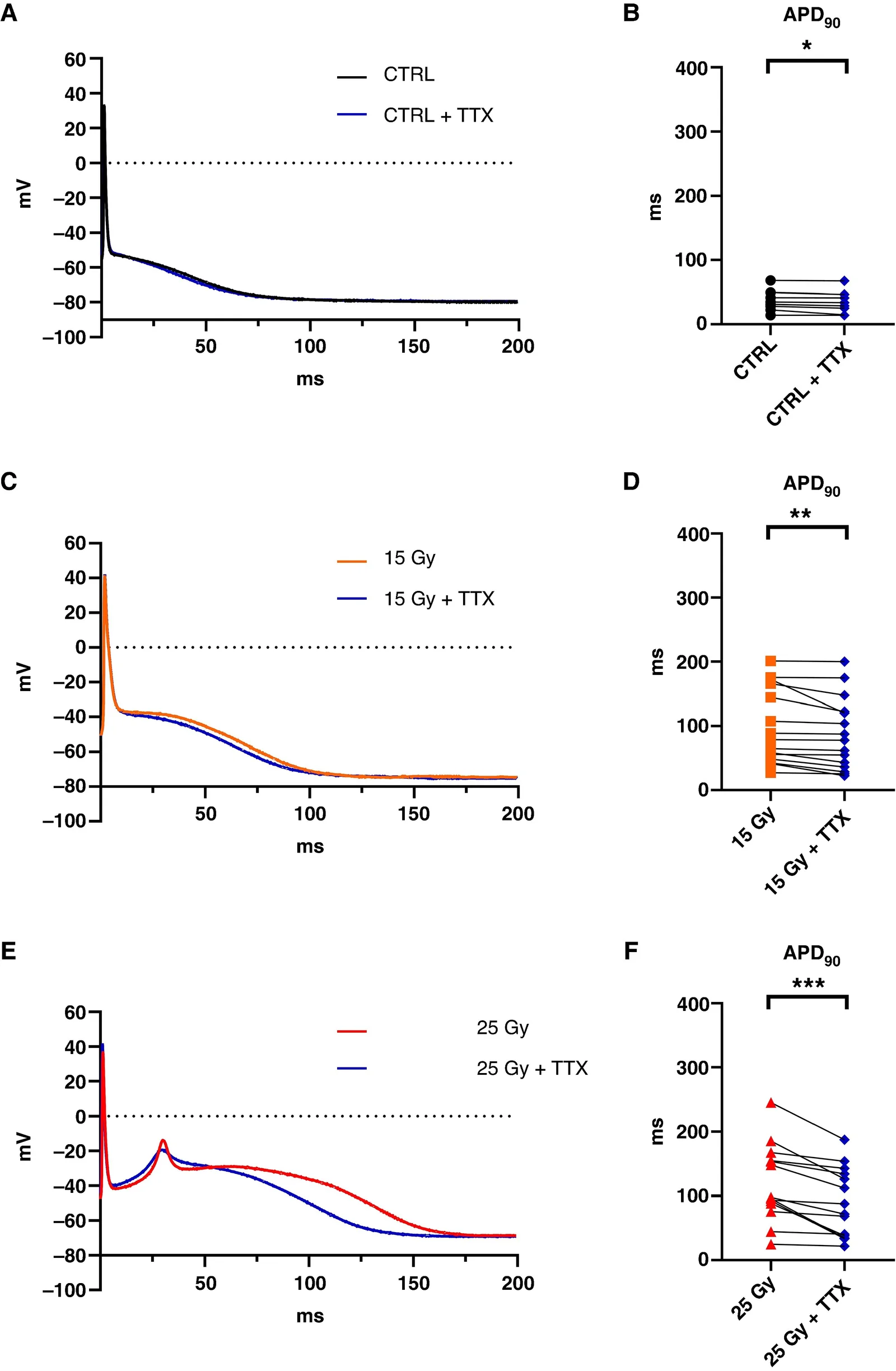
Effect of selective *I*_Nasus_ blockade (1 µM TTX) on APD_90_. (*A, C, E*) Representative traces of AP recorded during stimulation at 1 Hz. (*B, D, F*) APD90. CTRL: *n* = 8, *N* = 3; 15 Gy: *n* = 15, *N* = 3; 25 Gy: *n* = 14, *N* = 3. Data are expressed as mean ± SEM. Paired Student's *t-*test, *P < 0.05, **P < 0.01, ***P < 0.001, ****P < 0.0001.

Overall, irradiation modified AP parameters in a way fully compatible with the observed increments in *I*_NaT_ and *I*_Nasus_. The substantial APD prolongation may represent a further mechanism supporting VT suppression by RT.

### Effect of RT on intracellular Ca^2+^ dynamics

Since Na^+^ influx through *I*_Nasus_ affects intracellular Ca^2+^ handling, we evaluated the effect of RT on cytosolic Ca^2+^ dynamics in field-stimulated CMs.

#### Steady-state stimulation (1 Hz)

CaT parameters were quantified during steady-state stimulation at 1 Hz (*Figure 4*). In 25 Gy-irradiated CMs, CaT amplitude was significantly reduced (−56% *P* < 0.0001, *Figure 4C*) while CaD remained unchanged (*Figure 4B*). CaT kinetics were generally slowed by RT, as demonstrated by the increase in *t*_peak_ (+10%, *P* < 0.01, *Figure 4D*), the reduction in *d*Ca/*dt*_MAX_ (−10% *P* < 0.05, *Figure 4E*) and the increase in τ decay (+20%, *P* < 0.05, *Figure 4F*). SR Ca^2+^ content (CaSR) was not changed by 25 Gy RT (CTRL: 0.63 ± 0.06 nM/pF × 10^−8^ vs. 25 Gy: 0.72 ± 0.06 nM/pF × 10^−8^, Supplementary material online, *Figure S3*). In 15 Gy-irradiated CMs, a similar trend in CaT parameters was observed, but the changes were smaller and did not achieve statistical significance (*Figure 4*).

**Figure 4:**
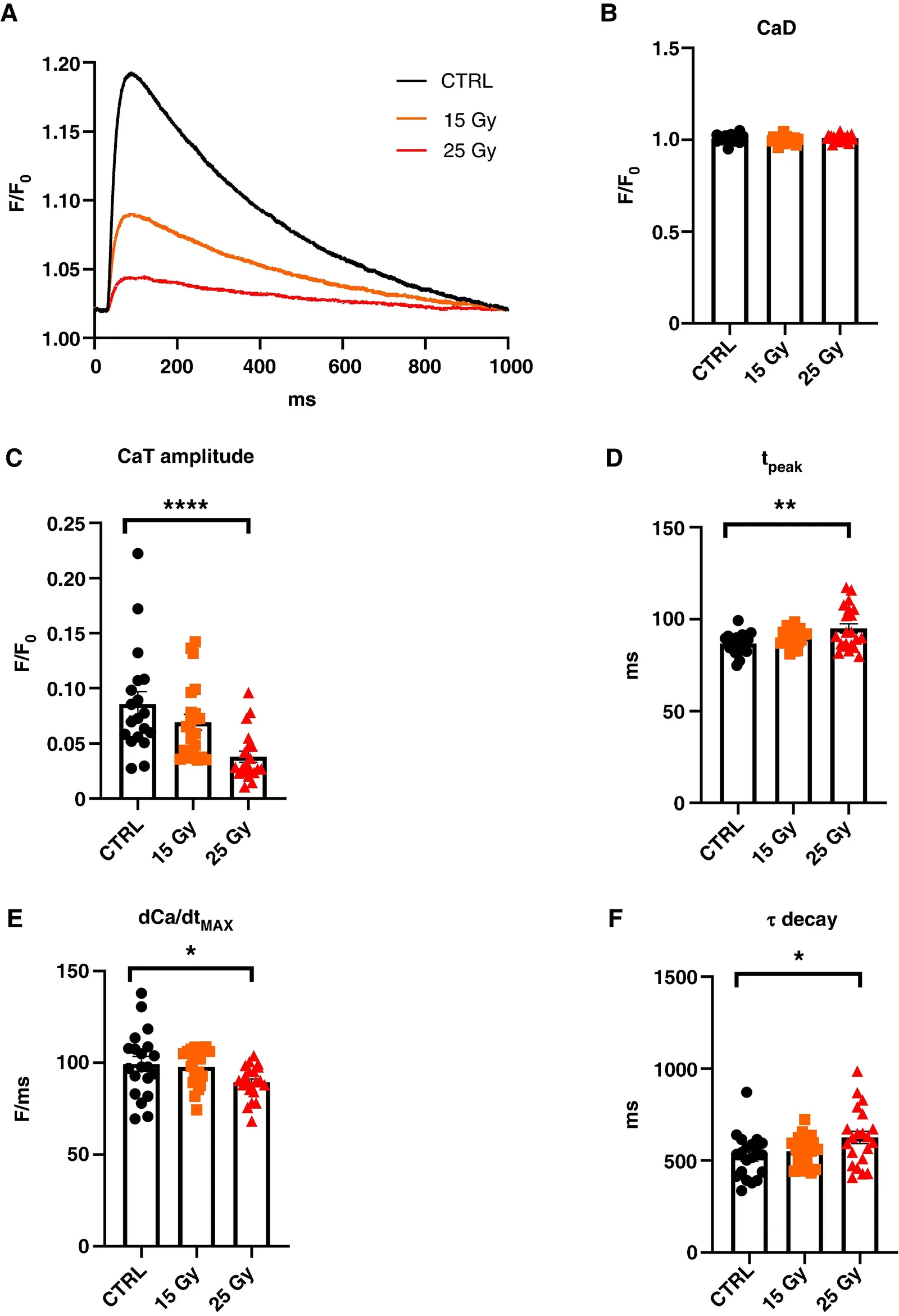
Effect of 15 and 25 Gy RT on Ca^2+^ transients (CaT) during steady-state stimulation at 1 Hz. (*A*) Representative CaT traces. (*B*) Diastolic Ca^2+^ (CaD). (*C*) CaT amplitude. (*D*) CaT time to peak (*t*_peak_). (*E*) Maximal rate of CaT rise (*d*Ca/*dt*_MAX_). (*F*) CaT decay kinetics (τ decay). CTRL: *n* = 20, *N* = 2; 15 Gy: *n* = 22, *N* = 3; 25 Gy: *n* = 21, *N* = 2. Data are expressed as mean ± SEM. One-way ANOVA followed by Dunnett's *post hoc* test (*B*, *D*, and *E*) and Kruskal–Wallis followed by Dunn's *post hoc* test (*C* and *F*), *P < 0.05, **P < 0.01, ***P < 0.001, ****P < 0.0001.

Overall, the results suggest a general downregulation of excitation-contraction (EC) coupling, compatible with simultaneous impairment of various among its components.

#### Rate-dependency

Rate-dependency of CaT properties was tested in a subset of cells by incrementing the pacing rate from 1 to 5 Hz (see Supplementary material online, *Figure S4*).

Under control conditions, CaT parameters showed significant rate-dependency (positive or negative): CaD (positive, *P* < 0.0001), *d*Ca/*dt*_MAX_ (positive, *P* < 0.0001), CaT amplitude (negative, *P* < 0.001), *t*_peak_ (negative, *P* < 0.0001), and τ_decay_ (negative, *P* < 0.0001).

As compared to CTRL, in 25 Gy CMs: CaT amplitude had a shallower rate-dependency (Treatment × Rate, *P* < 0.05) due to preferential reduction at slower rates; τ_decay_ had a steeper rate-dependency (Treatment × Rate, *P* < 0.05) due to preferential increase at slower rates. As expected from *I*_Nasus_ enhancement, CaD accumulation at a high rate tended to be more prominent in irradiated CMs. For *t*_peak_ and *d*Ca/*dt*_MAX_, rate-dependency remained unchanged between CTRL and 25 Gy.

With 15 Gy irradiation, the same trends were observed.

### Mechanism for *I*_Nasus_ enhancement

#### Evaluation of CaMKII phosphorylation and oxidation

The results so far indicate that 25 Gy RT enhanced *I*_Nasus,_ thus prolonging APD and altering the intracellular Ca^2+^ dynamics. In order to investigate the mechanisms for *I*_Nasus_ enhancement, we first quantified CaMKII activation by measuring its total and phosphorylated (pCaMKII) fractions by western blotting (*Figure 5*). In RT CMs, the pCaMKII/CaMKII ratio showed a dose-dependent trend to decrease, with a significant reduction at the 25 Gy dose (*Figure 5B*), which rules out CaMKII activation as a factor in *I*_Nasus_ enhancement. The oxiCaMKII/CaMKII ratio was not significantly changed by RT at all doses; nonetheless, a small dose-dependent increase in the mean value was consistently observed across triplicate measurements (see Supplementary material online, *Figure S5*).

**Figure 5:**
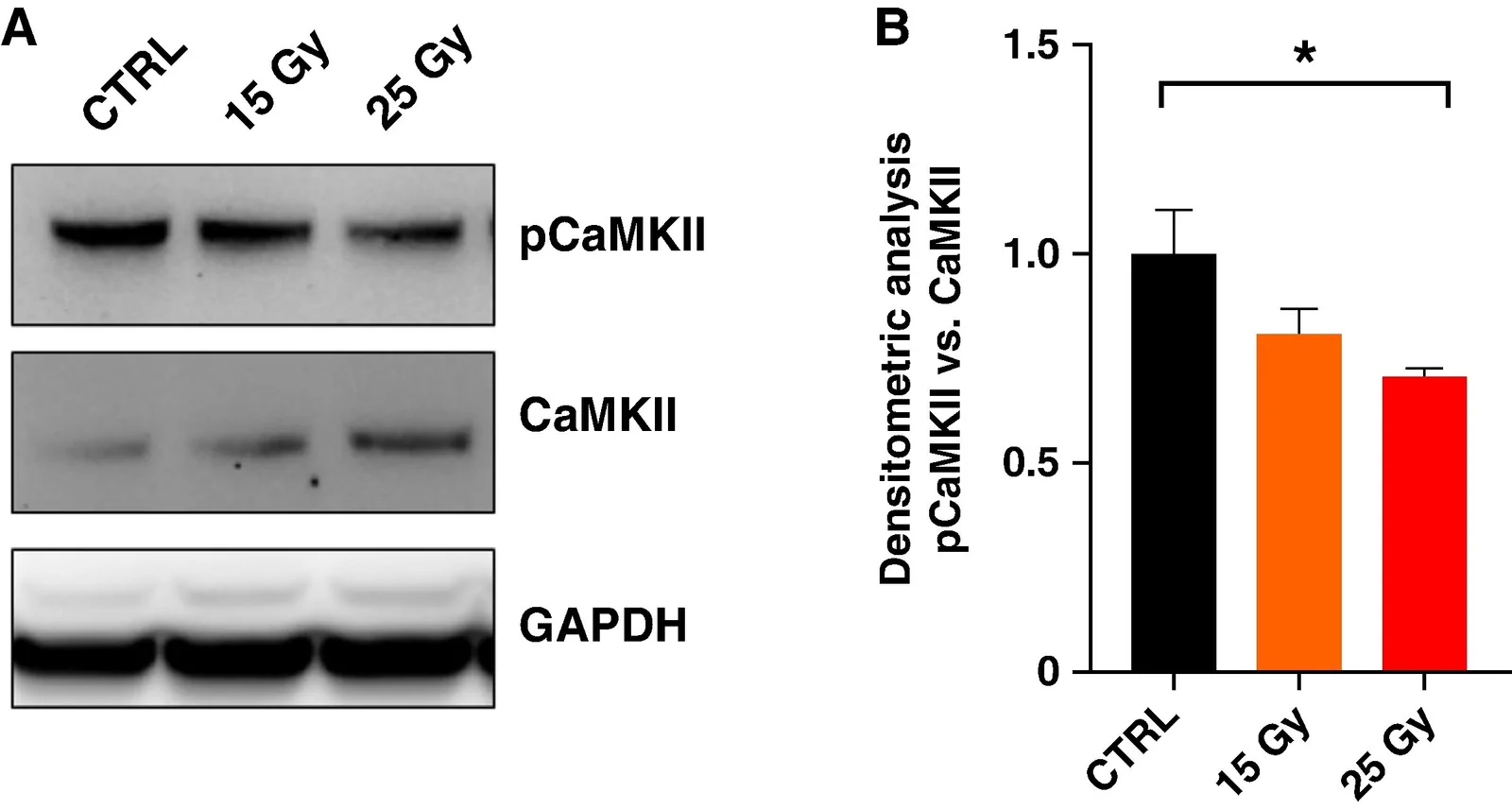
Protein analysis in cell pellet from ventricles. (*A*) Representative images of western Blot analysis of proteins extracted from ventricular tissue. Immunostaining of the housekeeping GAPDH is shown for normalization of total CaMKII. (*B*) Densitometric analysis of the pCaMKII/CaMKII ratio. CTRL: *N* = 3; 15 Gy, *N* = 3; 25 Gy: *N* = 3. Data expressed as mean ± SEM. One-way ANOVA followed by Dunnett's *post hoc* test, *P < 0.05.

#### Evaluation of cell ROS content

The intracellular content of ROS was estimated in quiescent CMs by measuring DCFDA fluorescence from individual vital cells (identified by the rod-shaped contour). The analysis was limited to the 25 Gy dose, the one consistently exerting significant effects on functional parameters (above). In 25 Gy CMs, DCFDA signal was significantly stronger than in CTRL CMs (+43%, *P* < 0.0001, *Figure 6B*), to indicate an increase in ROS content, a likely cause of *I*_Nasus_ enhancement and, possibly, depression of EC coupling.

**Figure 6:**
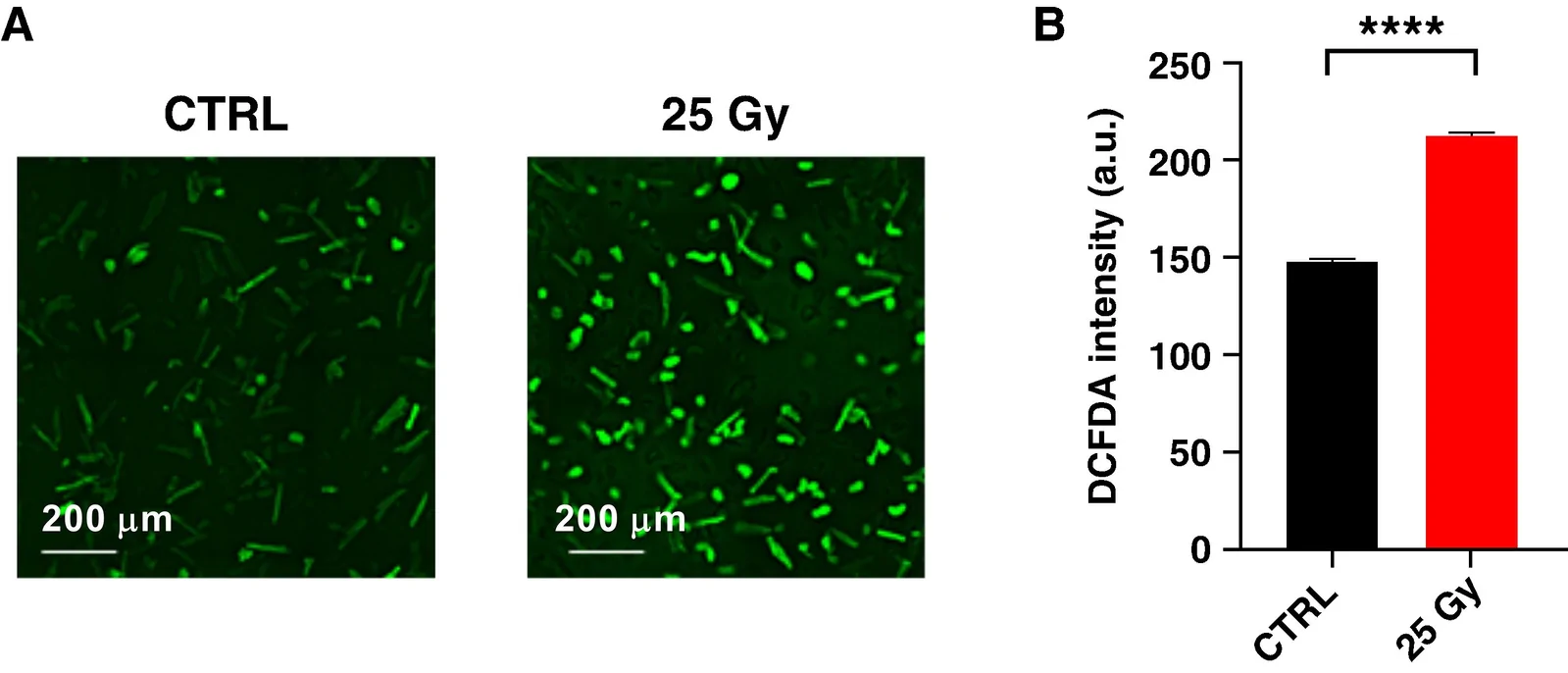
ROS quantification. (*A*) Fluorescence microscopic image (×20) of CMs incubated with 10 µM DCFDA. (*B*) DCFDA fluorescence intensity. CTRL: *N* = 3; 25 Gy: *N* = 3. Data expressed as mean ± SEM. Unpaired Student's *t-*test, ****P < 0.0001.

## Discussion

The results of this study show, by direct *I*_Na_ measurement, that a single myocardial irradiation at 25 Gy may substantially increase the activity of V-gated Na^+^ channels, previously inferred from the increase in the expression of NaV1.5 protein levels.^10^ They also show that the effect concerns both the transient and sustained *I*_Na_ components (*I*_NaT_ and *I*_Nasus_, respectively), with a preferential effect on the latter. An increase in ROS content provides a likely mechanism for selective *I*_Nasus_ increment. Irradiation-induced *I*_Na_ changes translate into significant APD prolongation and EADs facilitation. Irradiation was also associated with significant perturbation of intracellular Ca^2+^ handling; however, its direction is only partly consistent with what expected from increased Na^+^ influx and prolonged repolarization.

### *I*_Na_ modulation

The observed changes in overall *I*_Na_ are compatible with a radiation-induced increment in membrane expression of functional NaV1.5 channels^10^; however, the latter should increase the transient and sustained components of the current by a similar proportion. The observation of preferential *I*_Nasus_ enhancement suggests an additional effect of irradiation. Peroxidation of NaV1.5 channels by ROS is a well-established mechanism for enhancement of ‘late’ *I*_Na_ (*I*_NaL_),^17^ the ‘pathological’ contributor to *I*_Nasus_.^11^ Two observations suggest that *I*_NaL_ may account for the observed *I*_Nasus_ increment: (i) after irradiation maximal *I*_Nasus_ was achieved at more positive potentials, at which I_NaL_ prevails over the ‘window’ component; (ii) ROS content was indeed increased by irradiation. *I*_NaL_ enhancement in cardiac myocytes has also been attributed to increased expression of neuronal NaV isoforms characterized by incomplete inactivation.^18^ Since irradiation has been shown to affect channel transcription,^10^ such a mechanism would be plausible; however, the observation of a clear-cut increase in ROS points instead to post-transcriptional modulation of NaV1.5 proteins. RT weakened the correlation between *I*_NaT_ amplitude and *C*_m_, as implicit in the increase in *I*_NaT_ density. That *I*_Nasus_ amplitude was instead independent of *C*_m_ suggests that post-transcriptional modulation contributed to enhance this component beyond what justified by the sole increment in NaV1.5 expression. At any rate, since mean *C*_m_ did not differ among the experimental groups, the changes in current density among them do not depend on cell size.

### AP modulation

As shown by its sensitivity to *I*_Na_ blockade, repolarization slowing by irradiation is attributable to *I*_Nasus_ enhancement. APD prolongation may contribute to the effect of irradiation in contrasting ways: (i) by prolonging refractoriness it increases the wavelength of re-entrant circuits; along with the increment in conduction velocity, this may contribute to re-entry interruption, i.e. the desired effect of irradiation; (ii) by facilitating EADs it may increase the arrhythmogenic potential of the irradiated area.^19^ Of course, the effect of *I*_Na_ blockade does not rule out the contribution of currents other than *I*_Na_ to AP modulation by RT. Notably, APD was reportedly shortened 4 days after 20 Gy RT applied to cultured neonatal rat myocytes, consistent with overexpression of K^+^ channels.^20^  *I*_Na_ was unfortunately not measured in that study and the reason for this discrepancy remains elusive. Nonetheless, the absorbed RT energy (e.g. 25 Gy) is an estimate, its actual value might differ between a myocyte monolayer and closed chest irradiation, possibly explaining the difference.

EADs facilitation by irradiation may simply result from repolarization slowing,^21^ but it might also reflect spontaneous Ca^2+^ release event promoted by ROS-induced facilitation of RyRs opening.^22^ Indeed, the observation of a perturbed Ca^2+^ handling not entirely consistent with enhanced Na^+^ influx (see below) may suggest an additional (possibly ROS-mediated) radiation effect including destabilization of the Ca^2+^ store.

### Ca^2+^ handling perturbation

By increasing Na^+^ influx, *I*_Nasus_ enhancement is expected to hamper Ca^2+^ extrusion, thus increasing overall intracellular CaSR. This should lead to an increment in CaD and, unless associated with major destabilization of the Ca^2+^ stores, to larger CaT amplitude.

While a trend to rate-dependent CaD accumulation was indeed observed in irradiated CMs (*Figure 4B*), CaT amplitude was conspicuously reduced in a dose-dependent fashion, achieving statistical significance only at 25 Gy. Interestingly, this effect occurred without alterations in the CaSR. This suggests that Ca^2+^ handling was perturbed by irradiation also independently of *I*_Nasus_ enhancement. The function of practically all the elements of the Ca^2+^ handling machinery can be affected by peroxidation.^23^ Therefore, *I*_Nasus_ enhancement and Ca^2+^ handling perturbation might result from the increment in cell ROS content as parallel effects, rather than being linked in a causative chain. Notably, RyRs peroxidation is a well-established mechanism of EADs facilitation.^22^ In previous work, after 20 Gy RT applied to isolated mouse myocytes Ca^2+^ handling changed in a biphasic fashion: hyperdynamic at 1 h and depressed at 1 week post RT.^24^ Both these changes are compatible with RyRs facilitation, initially enhancing release, but ultimately leading to a leaky SR. These observations would be consistent with our findings (at 2 weeks), except for the preservation of CaSR in our case, which argues against a leaky SR as the mechanism of CaT depression.

### Increased ROS content

Redox imbalance is a known effect of ionizing radiations^25^; therefore, the observed increment in ROS content is unsurprising. Such increment was observed 15 days after the treatment, thus qualifying as a rather sustained action, possibly accounting for irradiation effects beyond the increment in *I*_Na_ density.

### Decreased CaMKII activation

The observed reduction in the pCaMKII/CaMKII ratio at 25 Gy might be the consequence of the reduced amplitude of the CaT, possibly complemented by ROS-mediated CaMKII oxidation. At any rate, RT changed pCaMKII in a direction opposite to that required to account for *I*_NaL_ enhancement.

### Dose-dependency of irradiation effects

All parameters were affected in the same direction by irradiations at 25 and 15 Gy with a clear-cut dose-dependency. Therefore, failure to achieve statistical significance of the 15 Gy effects on some parameters should not be necessarily interpreted as a lack of effect but rather as a consequence of their smaller magnitude. This view is supported by the observation that, albeit not achieving significance, the *I*_NaT_ increment induced by 15 Gy was associated with increments of APA and +*dV*/*dt*_MAX_ means, as expected from their dependency on *I*_NaT_ amplitude. On the other hand, 15 Gy irradiation did significantly increase *I*_Nasus_ and prolong APD_90_, thus suggesting higher sensitivity of these two parameters. Although the *I*_Nasus_ increment may be considered an untoward effect, APD_90_ prolongation would still support VT interruption. Overall, albeit less effective than 25 Gy, the 15 Gy dose might still exert biological effects compatible with discontinuation of re-entrant circuits but of potential proarrhythmic risk.

### Relevance to STAR therapy

As discussed above, *I*_NaL_ enhancement, EADs facilitation and depression of EC coupling may represent untoward effects of irradiation. The issue is to which extent these effects may dispute the therapeutic use of STAR.

The impact on overall cardiac function of the abnormalities disclosed by the present results may depend on the mass of muscle affected by the irradiation applied in the STAR procedure. Based on recent EHRA/HRS consensus data,^26^ clinically used STAR plans report median central target volumes (CTV, receiving the irradiation dose) of ∼35–50 mL and, once motion-management margins (5–10 mm) are considered, a peripheral target volume (PTV, including the tissue receiving scattered radiation) of ∼80–90 mL. Thus, in the case of a 25 Gy dose, a CTV of ∼40–50 mL receives 25 Gy and an additional ∼40–50 mL of tissue surrounding the CTV is exposed to radiation doses <25 Gy, tapering with distance.

Considering a muscle density of 1.06 g/mL a CTV of 50 mL corresponds to 53 g of muscle mass (≅ 50% of the left ventricular mass index in male), and an even larger mass is included in the PTV. The irradiated myocardium (whose excitability is enhanced) might become a source of EAD-dependent propagated activity and/or steep repolarization gradients, thereby representing a proarrhythmic risk. Such risk would depend on the affected myocardial mass, thus making beam focusing of pivotal importance.

While VT prevention (by conduction boost) may depend on *I*_NaT_ upregulation by transcriptional reprogramming,^10^ the proarrhythmic abnormalities may be linked to *I*_NaL_ enhancement instead, to which ROS may contribute significantly. If the oxidative response to irradiation were relatively short-lived, this might explain the frequent VT recurrence during the ‘blanking period’ (6 weeks after STAR), which may subside in the subsequent months.^6^ Notably, the mechanism underlying post-procedure VT exacerbation might theoretically differ from that of the VT to be treated.

*I*_NaL_ can be selectively blocked by suitable class I antiarrhythmic agents (e.g. mexiletine, ranolazine, amiodarone), which would remove the associated risk but, at the same time, prevent APD prolongation. Should the latter contribute significantly to discontinuation of the re-entrant circuit responsible for VT, a plausible but unproven hypothesis, the corrective measure might theoretically limit STAR efficacy in VT treatment.

Currently, myocardial conduction can be improved only through gene-therapy approaches of difficult clinical applicability. Because of its effect on *I*_NaT_, irradiation might have an appeal as a putative treatment of conditions characterized by a diffuse conduction impairment (e.g. loss of function NaV mutations, infarct border zone, etc.). However, in this case irradiation should be extended to even larger tissue volumes, potentially aggravating the side effects disclosed by the present study.

### Limitations

The conditions required for simultaneous measurement of *I*_NaT_ and *I*_Nasus_ within the same myocyte resulted in large *I*_NaT_ amplitudes, for which the errors introduced by series resistance and capacitance might be significant, both potentially leading to underestimate RT-induced *I*_NaT_ increment. The estimated *V* error for the average *I*_NaT_ increment at 25 Gy (2.6 nA) is small (2.6 mV); nonetheless, it might lead to slightly overestimate RT-induced changes in the *I*_Nasus_/*I*_NaT_ ratio.

While RT is mostly applied to the treatment of VT in the presence of structural heart disease, the present results have been obtained in healthy hearts. On the other hand, non-ablative VT interruption, for which *I*_NaT_ enhancement is the most plausible explanation, has been observed in diseased hearts. *I*_Nasus_ enhancement could be even more relevant in the latter, often intrinsically burdened by redox imbalance.

Only male animals were studied with the aim of ruling out sex as a source of variability. Nonetheless, this should be considered in generalization of results.

Comparison with previous results obtained at different time-points after RT suggests that the effect of a single RT application may change over time; therefore, long-term studies may be necessary to assess the stable consequences of RT on cell physiology.

## Conclusions

The effects of a single irradiation dose of 25 Gy on *I*_NaT_ are consistent with the increased expression of NaV1.5 protein and account for the improvement of conduction.^10^ By prolonging APD, simultaneous *I*_NaL_ enhancement may provide an additional mechanism for VT interruption, but it may render the (still excitable) irradiated myocardium a source of ectopic activity and weakened contraction. This may require consideration in the therapeutic application of highly focused irradiation (STAR) but might establish a contraindication to the potential use of more diffuse cardiac irradiation.

## Supplementary Material

euag282_Supplementary_Data
