# Supplementary material for: Cellular consequences of non-ablative radiotherapy: A novel approach to ventricular tachycardias
Source: Europace. 2026 Oct 17;28(10):euag282. doi: 10.1093/europace/euag282 (PMC13712131; doi:10.1093/europace/euag282)
Supplement: Supplementary file 1 [file euag282_supplementary_data.docx]

**SUPPLEMENT**

**DETAILED MATERIALS AND METHODS**

**Cardiomyocyte isolation**

All animal procedures were conducted in accordance with Milano-Bicocca animal care guidelines and the European Directive 2010/63/EU on the protection of animals used for scientific purposes.

Mice were intraperitoneally anesthetized with mixture of 20 mg of ketamine-xylazine (130–7.5 mg/kg i.p), 200 μL of Seleparin (Nadroparin Calcium 9500 U.I, ItalFarmaco) and 200 μL of saline (0.9% NaCl 1M in MilliQ), euthanized by cervical dislocation. Ventricular cardiomyocytes (CMs) were isolated using a manual perfusion technique^1^ which does not discriminate between right and left ventricles. Briefly, while the heart was in situ, the right ventricle was perfused with an EDTA-based buffer (130 mM NaCl, 5 mM KCl, 0.5 mM NaH_2_PO_4_, 10 mM HEPES, 10 mM Glucose, 10 mM 2,3-Butanedione Monossime, BDM, 10 mM Taurine, 5 mM EDTA; pH=7.8 with NaOH), after which the aorta was clamped and the heart removed. Enzymatic digestion was performed by sequential perfusion of the left ventricle with EDTA buffer, perfusion buffer (130 mM NaCl, 5 mM KCl, 0.5 mM NaH_2_PO_4_, 10 mM HEPES, 10 mM Glucose, 10 mM 2,3-Butanedione Monossime, BDM, 10 mM Taurine, 1 mM MgCl_2_; pH=7.8 with NaOH), and a Liberase/trypsin solution (0.1 mg/ml Liberase + 0.02 mg/ml Trypsin EDTA and 6.25 µM CaCl_2_). Cells were then gently dissociated, and enzymatic activity was stopped with a serum-containing buffer (10% FBS). The cell suspension was filtered, centrifuged, resuspended in calcium-free solution (130 mM NaCl, 5.4 mM KCl, 0.4 mM NaH_2_PO_4_, 0.5 mM MgCl_2_, 25 mM Hepes, 22 mM Glucose; pH=7.4 with NaOH). Before the experiments, physiological extracellular Ca^2+^ levels (2 mM) were gradually restored.

**Protein extraction and western blot analysis**

CMs were lysed in cell lysis buffer (Cell Signaling Technology, Danvers, MA, USA) supplemented with protease and phosphatase inhibitor cocktails (Sigma-Aldrich, Saint Louis, MO, USA). Total protein extracts were subjected to SDS-PAGE and transferred onto a nitrocellulose membrane (Bio-Rad, California, USA). The membranes were blocked for 1 h at room temperature in 5% non-fat dry milk in Wash Buffer (Tris Buffer Sulfate, 0.1% Tween-20) and then incubated O/N at 4 °C with the appropriate primary antibodies (reported in **Table S1**). The membranes were incubated with peroxidase-conjugated secondary antibodies (GE Healthcare, Chicago, IL, USA) for 1 h. Signals were visualized using the LiteUP Western Blot Chemiluminescent Substrate (EuroClone, Milan, Italy). Images were acquired with the ChemiDoc™ MP Imaging System (Bio-Rad, California, USA), and densitometric analysis of membranes was performed using the ImageJ software (National Institutes of Health, Bethesda, MD, USA). CMs CaMKII levels were normalized to GAPDH.

| **Protein** | **Clonality/Code** | **Source** | **Company** | **Diluition** |
| --- | --- | --- | --- | --- |
| phospho-CaMKII (T286) | Polyclonal, ab32678 | Rabbit | Abcam | WB: 1:1000 |
| oxidized-CaMKII  (Met281/282) | Polyclonal, 07-1387 | Rabbit | Millipore | WB: 1:1000 |
| CaMKII | Monoclonal, sc-5306 | Mouse | Santa Cruz | WB: 1:500 |
| GAPDH | Polyclonal, sc-25778 | Rabbit | Santa Cruz | WB: 1:1000 |

**Table S1.** Primary antibodies.

**SUPPLEMENTARY RESULTS**

|  | **CTRL** | **15 Gy** | **25 Gy** |
| --- | --- | --- | --- |
| C_m_ (pF) | 211 ± 9 (n=18) | 218 ± 14 (n=17) | 212 ± 6 (n=16) |
| I_NaT_ (pA) | -3905 ± 593 (n=18) | -4490 ± 455 (n=17) | -6519 ± 1000 (n=16) |
| I_NaT_ (pA/pF) | -18.43 ± 2.47 (n=18) | -20.69 ±1.82 (n=17) | -30.39 ± 4.53 (n=16) |
| τ_h_ (ms) | 0.38 ± 0.01 (n=18) | 0.41 ± 0.01 (n=17) | 0.40 ± 0.02 (n=16) |
| Mean I_Nasus_ (pA) | -93 ± 9 (n=18) | -166 ± 19 (n=17) | -324 ± 31 (n=16) |
| Mean I_Nasus_ (pA/pF) | -0.45 ± 0.05 (n=18) | -0.79 ± 0.09 (n=17) | -1.53 ± 0.14 (n=16) |
| I_Nasus_/I_NaT_ (%) | 1.32 ± 0.14 (n=18) | 2.55 ± 0.27 (n=17) | 4.39 ± 0.55 (n=16) |
| E_rev_ | 45.62 ± 1.89 (n=18) | 46.68 ± 2.40 (n=17) | 43.71 ± 2.38 (n=16) |

**Table S2**. Summary of I_NaT_ and I_Nasus_ values.

|  | **CTRL** | **15 Gy** | **25 Gy** |
| --- | --- | --- | --- |
| APA (mV) | 101 ± 4.33 (n=16) | 109 ± 2.11 (n=17) | 113 ± 3.34 (n=19) |
| APD_90_ (ms) | 55.19 ± 7.27 (n=16) | 93.12 ± 13.71 (n=17) | 116 ± 16.79 (n=19) |
| +dV/dt_MAX_ (mV/ms) | 222 ± 27.09 (n=16) | 255 ± 15.34 (n=17) | 307 ± 27.68 (n=19) |
| E_diast_ (mV) | -77.01 ± 1.81 (n=16) | -80.39 ± 1.43 (n=17) | -74.93 ± 1.42 (n=19) |

**Table S3.** Summary of AP parameters.

|  | **CTRL** | **TTX** | **15 Gy** | **TTX** | **25 Gy** | **TTX** |
| --- | --- | --- | --- | --- | --- | --- |
| APA (mV) | 109 ± 5.81 (n=8) | 104 ± 5.86  (n=8) | 110 ± 1.94 (n=15) | 106 ± 2.15 (n=15) | 114 ± 3.74 (n=14) | 108 ± 4.82 (n=14) |
| APD_90_ (ms) | 36.22 ± 5.97  (n=8) | 33.93 ± 6.27 (n=8) | 98.23 ± 15.07 (n=15) | 87.16 ± 14.55 (n=15) | 119 ± 15.84 (n=14) | 89.55 ± 14.26 (n=14) |
| +dV/dt_MAX_ (mV/ms) | 280 ± 37.12 (n=8) | 238 ± 38.85 (n=8) | 264 ± 15.59  (n=15) | 237 ± 16.81 (n=15) | 316 ± 36.95 (n=14) | 251 ± 34.54 (n=14) |
| E_diast_ (mV) | -79.76 ± 2.73  (n=8) | -76.62 ± 3.09 (n=8) | -80.46 ± 1.49 (n=15) | -80.22 ± 1.54 (n=15) | -75.69 ± 1.53  (n=14) | -76.61 ± 1.71 (n=14) |

**Table S4.** Summary of AP parameters before and after selective I_NaL_ blockade (1 μM TTX).

|  | **CTRL** | **15 Gy** | **25 Gy** |
| --- | --- | --- | --- |
| CaD (F/F0) | 1.008 ± 0.004 (n=20) | 1.003 ± 0.004 (n=21) | 1.009 ± 0.004 (n=21) |
| CaT amplitude (F/F0) | 0.086 ± 0.011 (n=19) | 0.069 ± 0.007 (n=22) | 0.038 ± 0.005 (n=20) |
| tpeak (ms) | 86.75 ± 1.233 (n=20) | 90.02 ± 1.011 (n=21) | 95.04 ± 2.547 (n=21) |
| dCa/dt_MAX_ (F/ms) | 99.35 ± 4.033 (n=20) | 96.04 ± 2.616 (n=22) | 89.28 ± 1.949 (n=21) |
| τdecay (ms) | 523 ± 27.193 (n=20) | 551 ± 16.391 (n=22) | 625 ± 33.891 (n=21) |

**Table S5.** Summary of CaT parameters during steady-state stimulation at 1 Hz.

|  | **CTRL** | **15 Gy** | **25 Gy** |
| --- | --- | --- | --- |
| CaD (F/F0)_1Hz | 1.008 ± 0.004 (n=20) | 1.003 ± 0.004 (n=21) | 1.008 ± 0.004 (n=19) |
| CaD (F/F0)_5Hz | 1.049 ± 0.007 | 1.069 ± 0.008 | 1.078 ± 0.014 |
| CaT amplitude (F/F0)_1 Hz | 0.086 ± 0.011 (n=19) | 0.069 ± 0.007 (n=22) | 0.047 ± 0.010 (n=19) |
| CaT amplitude (F/F0)_5 Hz | 0.061 ± 0.006 | 0.052 ± 0.005 | 0.044 ± 0.009 |
| tpeak (ms)_1 Hz | 86.75 ± 1.233 (n=20) | 90.02 ± 1.011 (n=21) | 94.058 ± 2.575 (n=19) |
| tpeak (ms)_5 Hz | 59.34 ± 0.939 | 60.89 ± 1.708 | 66.82 ± 3.222 |
| dCa/dt_MAX_ (F/ms)_1 Hz | 99.35 ± 4.033 (n=20) | 96.04 ± 2.616 (n=22) | 89.55 ± 2.049 (n=19) |
| dCa/dt_MAX_ (F/ms)_5 Hz | 119 ± 4.116 | 122 ± 4.032 | 100 ± 5.759 |
| τdecay (ms)_1 Hz | 523 ± 27.193 (n=20) | 551 ± 16.391 (n=22) | 612 ± 35.683 (n=19) |
| τdecay (ms)_5 Hz | 384 ± 27.868 | 388 ± 32.047 | 359 ± 37.425 |

**Table S6.** Summary of CaT parameters during increasing stimulation frequency (from 1 Hz to 5 Hz).

|  | **CTRL** | **15 Gy** | **25 Gy** |
| --- | --- | --- | --- |
| pCaMKII vs CaMKII | 1 ± 0.106 (N=3) | 0.808 ± 0.006 (N=3) | 0.707 ± 0.018 (N=3) |
| oxCaMKII vs CaMKII | 1 ± 0.210 (N=3) | 1.024 ± 0.195 (N=3) | 1.182 ± 0.088 (N=3) |

**Table S7.** Quantification by densitometric analysis of the pCaMKII/CaMKII and oxiCaMKII/CaMKII ratio.

|  | **CTRL** | **25 Gy** |
| --- | --- | --- |
| ROS  (DCFDA fluorescence intensity, a.u.) | 148 ± 1.442  (n=20231) | 213 ± 2.067  (n=25356) |

**Table S8.** DCFDA fluorescence intensity.

**Figure S1. Correlation between I_NaT_ and Cm**. Scatter plots showing the relationship between I_NaT_ and Cm in each experimental group (CTRL, 15 Gy, and 25 Gy). Linear regression analysis (red line) with the corresponding P value and coefficient of determination (R²) are reported. CTRL: n=18, N=3; 15 Gy: n=17, N=5; 25 Gy: n=16, N=2.

**Figure S2. Correlation between I_Nasus_ and Cm**. Scatter plots with linear regression (red line), P value and R² (as for Fig. S1). CTRL: n=18, N=3; 15 Gy: n=17, N=5; 25 Gy: n=16, N=2.

**Figure S3. Effect of 25 Gy RT on SR Ca^2+^ load**. (**A**) Representative traces of membrane currents recorded during caffeine-induced SR release (representative of the current mediated by the Na/Ca exchanger, I_NCX)_. (**B**) SR Ca^2+^ content (CaSR) estimated from the integral of the traces in panel A. CTRL: n=12, N=3; 25 Gy: n=19, N=3. Data are expressed as mean ± SEM. Unpaired Student’s t test.

**Figure S4. Rate dependency of CaT parameters.** Rate-dependency was tested by incrementing the pacing rate from 1 to 5 Hz. (**B**) Diastolic Ca^2+^ (CaD). (**C**) CaT amplitude. (**D**) CaT time to peak (t_peak_). (**E**) Maximal rate of CaT rise (dCa/dt_MAX_). (**F**) CaT decay kinetics (τ decay). CTRL: n=20, N=2; 15 Gy: n=22, N=3; 25 Gy: n=19, N=2. Data are expressed as mean ± SEM. Two-way ANOVA followed by Dunnett’s post-hoc test, * p<0.05; ** p<0.01; the horizontal bars denote significance for the treatment X rate effect; the vertical brackets denote significance for the treatment effect.

**Figure S5. Protein analysis in cell pellet from ventricles**. (**A**) Representative images of Western Blot analysis of proteins extracted from ventricular tissue. Immunostaining of the housekeeping GAPDH is shown for normalization of total CaMKII. (**B**) Densitometric analysis of the oxCaMKII/CaMKII ratio. CTRL: N=3; 15 Gy, N=3; 25 Gy: N=3. Data expressed as mean ± SEM. One-way ANOVA followed by Dunnett’s post-hoc test.

**References**

1.	Ackers-Johnson M, Li PY, Holmes AP, O'Brien SM, Pavlovic D, Foo RS. A Simplified, Langendorff-Free Method for Concomitant Isolation of Viable Cardiac Myocytes and Nonmyocytes From the Adult Mouse Heart. *Circ Res* 2016;**119**:909-920.
